# Citations and metrics of journals discontinued from Scopus for publication concerns: the GhoS(t)copus Project

**DOI:** 10.12688/f1000research.23847.2

**Published:** 2020-08-26

**Authors:** Andrea Cortegiani, Mariachiara Ippolito, Giulia Ingoglia, Andrea Manca, Lucia Cugusi, Anna Severin, Michaela Strinzel, Vera Panzarella, Giuseppina Campisi, Lalu Manoj, Cesare Gregoretti, Sharon Einav, David Moher, Antonino Giarratano

**Affiliations:** 1 Department of Surgical, Oncological and Oral Science (Di.Chir.On.S.), University of Palermo, Department of Anesthesia Intensive Care and Emergency, Policlinico Paolo Giaccone, Palermo, Italy; 2 Department of Biomedical Sciences, University of Sassari, Sassari, 07100, Italy; 3 Swiss National Science Foundation, Swiss National Science Foundation, Bern, CH-3001, Switzerland; 4 Institute for Social and Preventive Medicine, Institute for Social and Preventive Medicine, Bern, 3012, Switzerland; 5 Department of Surgical, Oncological and Oral Science (Di.Chir.On.S), Section of Oral Medicine, University of Palermo, Palermo, 90127, Italy; 6 Centre for Journalology, Clinical Epidemiology Program, University of Ottawa, Ottawa, 201B, Canada; 7 Intensive Care Unit of the Shaare Zedek Medical Medical Centre, Hebrew University Faculty of Medicine, Jerusalem, Israel

**Keywords:** predatory, journal, Scopus, metrics, indexing, citation count

## Abstract

**Background:** Scopus is a leading bibliometric database. It contains a large part of the articles cited in peer-reviewed publications **. **The journals included in Scopus are periodically re-evaluated to ensure they meet indexing criteria and some journals might be discontinued for 'publication concerns'. Previously published articles may remain indexed and can be cited. Their metrics have yet to be studied. This study aimed ****to evaluate the main features and metrics of journals discontinued from Scopus for publication concerns, before and after their discontinuation, and to determine the extent of predatory journals among the discontinued journals.

**Methods:** We surveyed the list of discontinued journals from Scopus (July 2019). Data regarding metrics, citations and indexing were extracted from Scopus or other scientific databases, for the journals discontinued for publication concerns.

**Results:** A total of 317 journals were evaluated. Ninety-three percent of the journals (294/317) declared they published using an Open Access model. The subject areas with the greatest number of discontinued journals were *Medicine* (52/317; 16%), *Agriculture and Biological Science* (34/317; 11%), and *Pharmacology, Toxicology and Pharmaceutics *(31/317; 10%). The mean number of citations per year after discontinuation was significantly higher than before (median of difference 16.89 citations, p<0.0001), and so was the number of citations per document (median of difference 0.42 citations, p<0.0001). Twenty-two percent (72/317) were included in the Cabell’s blacklist. The DOAJ currently included only 9 journals while 61 were previously included and discontinued, most for 'suspected editorial misconduct by the publisher'.

**Conclusions:** Journals discontinued for 'publication concerns' continue to be cited despite discontinuation and predatory behaviour seemed common. These citations may influence scholars’ metrics prompting artificial career advancements, bonus systems and promotion. Countermeasures should be taken urgently to ensure the reliability of Scopus metrics for the purpose of scientific assessment of scholarly publishing at both journal- and author-level.

## Introduction

Scopus is a leading bibliometric database launched in 2004 by the publishing and analytics company Elsevier. It was developed by research institutions, researchers and librarians, and contains the largest number of abstracts and articles cited in peer reviewed academic journal articles that cover scientific, technical, medical, and social science fields ^1^.

Scopus provides bibliometric indicators that many institutions use to rank journals to evaluate the track record of scholars who seek hiring or promotion. These metrics are also used to allocate financial bonuses or to evaluate funding applications ^2– 4^. Ensuring the quality of the content of the Scopus database is therefore of great importance.

Scopus indexed journals undergo evaluation and periodic review by an independent and international Content Selection and Advisory Board (CSAB), a group of scientists, researchers and librarians, comprised of 17 Subject Chairs, each representing a specific subject field- and by a computerized algorithm ^1^. At any time after journal inclusion, concerns regarding its quality may be raised by a formal complaint, thereby flagging the journal for re-evaluation by the CSAB. Should the CSAB panel determine that the journal no longer meets Scopus standards, new articles from that journal are no longer indexed ^1^. One of the most common reasons for discontinuation is ‘publication concerns’, which refers to the quality of editorial practices or other issues that have an impact on its suitability for continued coverage ^5^. The list of the discontinued sources is publicly available and is updated approximately every six months ^6^. However, articles published in journals that were discontinued and are no longer indexed, are probably not removed from the Scopus database.

It has been claimed that a number of journals discontinued from Scopus for publication concerns might be so-called ‘predatory’ journals ^5^. Predatory journals “prioritize self-interest at the expense of scholarship and are characterized by false or misleading information, deviation from best editorial and publication practices, a lack of transparency, and/or the use of aggressive and indiscriminate solicitation practices” ^7^. Since researchers are pressured to publish in indexed journals, predatory journals are constantly trying to be indexed in the Scopus database, thereby boosting their attractiveness to researchers ^2, 8^. Having articles from predatory journals indexed in Scopus poses a threat to the credibility of science and might cause harm particularly in fields where practitioners rely on empirical evidence in the form of indexed journal articles ^8, 9^.

We hypothesize that, even though Scopus coverage is halted for discontinued journals, they are still cited, as all their documents, that are already indexed, remain available to users. To date, the metrics of those journals discontinued for publication concerns have not been studied yet. Therefore, in the present analysis we set out to (1) evaluate the main scientific features and citation metrics of journals discontinued from Scopus for publication concerns, before and after discontinuation, and (2) determine the extent of predatory journals included in the discontinued journals.

## Methods

### Search strategy

The freely accessible and regularly updated Elsevier list (see Source data) of journals discontinued from the Scopus database (version July 2019) ^10^ was accessed on 24 ^th^ January 2020 (See Underlying data ^11^). We restricted our analysis to journals discontinued for “publication concerns”. Journals were checked for relevant data (described below), which were then independently collected by four pairs of authors (MI and GI, AM and LC, AS and MS, VP and AC), each pair being assigned one quarter of the data to be collected in duplicate. The data were collected using a standardized data extraction form (Underlying data Table 1). A second check to confirm the data and resolve discrepancies was performed by four additional authors that had not been involved in data collection (LM, CG, SE, AG). Data collection was initiated on 24 ^th^ January and completed by the end of February 2020. Confirmed data were registered on an Excel datasheet ( *Underlying data*, Table 1 ^11^).

### Retrieved data and sources

Data were extracted either from the Scopus database ^10^ or by searching other sources, such as SCImago Journal & Country Rank (SJCR) ^12^, Journal Citation Reports ^13^, Centre for Science and Technology Studies (CWTS) Journal Indicators ^14^, Beall’s updated List ^15^, Directory of Open Access Journals (DOAJ) ^16^, PubMed ^17^ and Web of Science ^18^. Open Access policy was checked on journals websites. The standardized data extraction form, independently applied by eight authors (MI, GI, AM, LC, AS, MS, VP, AC), was used to collect the following data: journal title, name and country of the publisher, the number of years of Scopus coverage, year of Scopus discontinuation, subject areas and sub-subject areas, Impact Factor (IF), CiteScore, SCImago Journal Rank (SJR), Source Normalized Impact *per* Paper (SNIP), best SCImago quartile, the indexing of at least one article in PubMed, Web Of Science (WOS) and DOAJ (for open access journals) indexing, presence in the updated Beall’s List, total number of published documents and total number of citations. All the metrics were checked on the year of Scopus discontinuation. In cases of discrepancies between Scopus data and other sources, Scopus data was preferred.

We defined the ‘before discontinuation’ time frame as the period included within the first year of journal coverage by Scopus and the year of discontinuation, which was not included in our calculations. The ‘after discontinuation’ time frame, was defined as the period included within the year of Scopus discontinuation and 2020. If the journal had been discontinued more than once, the time frame was based on the last one, according to the date of the last document displayed in the Scopus database. Citations ‘before’ and ‘after’ the date of discontinuation were manually counted based on either the Scopus journal overview or the downloadable tables made available by Scopus upon request (see Source data). When evaluating the presence of articles in PubMed (e.g. PubMed Central) and WOS and DOAJ indexing, 2019 was considered the reference year, preventing disadvantages for journals with time gaps for publication.

We calculated the median number of cumulative citations across all discontinued journals per year of coverage and defined it as ‘Citations per year’. We also calculated the median number of cumulative citations across all discontinued journals per document (‘Citations per document’). We included all documents indexed in Scopus, regardless of type. Finally, one author (AS) checked whether discontinued journals were present in Cabell’s whitelist or blacklist ^19^ or the DOAJ’s list of discontinued journals ^20^. As some of the journals included in the blacklist lack ISSNs or other unique identifiers, the comparison of the three lists with Scopus’s discontinued journals was based on matching the journals’ names by similarity using the Jaro-Winkler algorithm in RStudio Desktop 1.2.5033 and RecordLinkage 0.4–11.2 following the approach developed by Strinzel *et al*. (2019) ^21, 22^. The Jaro-Winkler metric, scaled between 0 (no similarity) and 1 (exact match), was calculated for all possible journals’ pairings ^23^. We manually inspected all pairs with a Jaro-Winkler metric smaller than one in order to include cases where, due to the orthographical differences between the lists, no exact match was found. For each matched pair, we compared journal publishers and, where possible, ISSNs in order to exclude cases where two journals had the same or a similar name but were edited by different publishers.

Full definitions and descriptions of the sources and metrics are reported in the Extended Data Appendix 1 ^24^.

### Statistical analysis

All data management and calculations were performed using Microsoft Excel (version 2013, Microsoft Corporation®, USA) and GraphPad Prism (version 8.3.1, 322, GraphPad software®, San Diego California). Variable distribution was assessed for normality using the D’Agostino-Pearson test. For variables with normal distribution means and standard deviations (SDs) were reported. For non-normally distributed data medians, interquartile ranges (IQRs, 25th–75th) and ranges (minimum value - maximum value) were reported. Categorical data were expressed as proportions and percentages.

The paired sample *t* test or the Wilcoxon matched-pairs signed ranked test were used to compare journal data before and after Scopus discontinuation, as appropriate.

## Results

Data could be retrieved regarding 317 of the 348 journals listed as discontinued (91.1%). The remaining journals were not found on the Scopus database using the search tool.

### Journals’ and publishers’ characteristics

**Figure 1.**
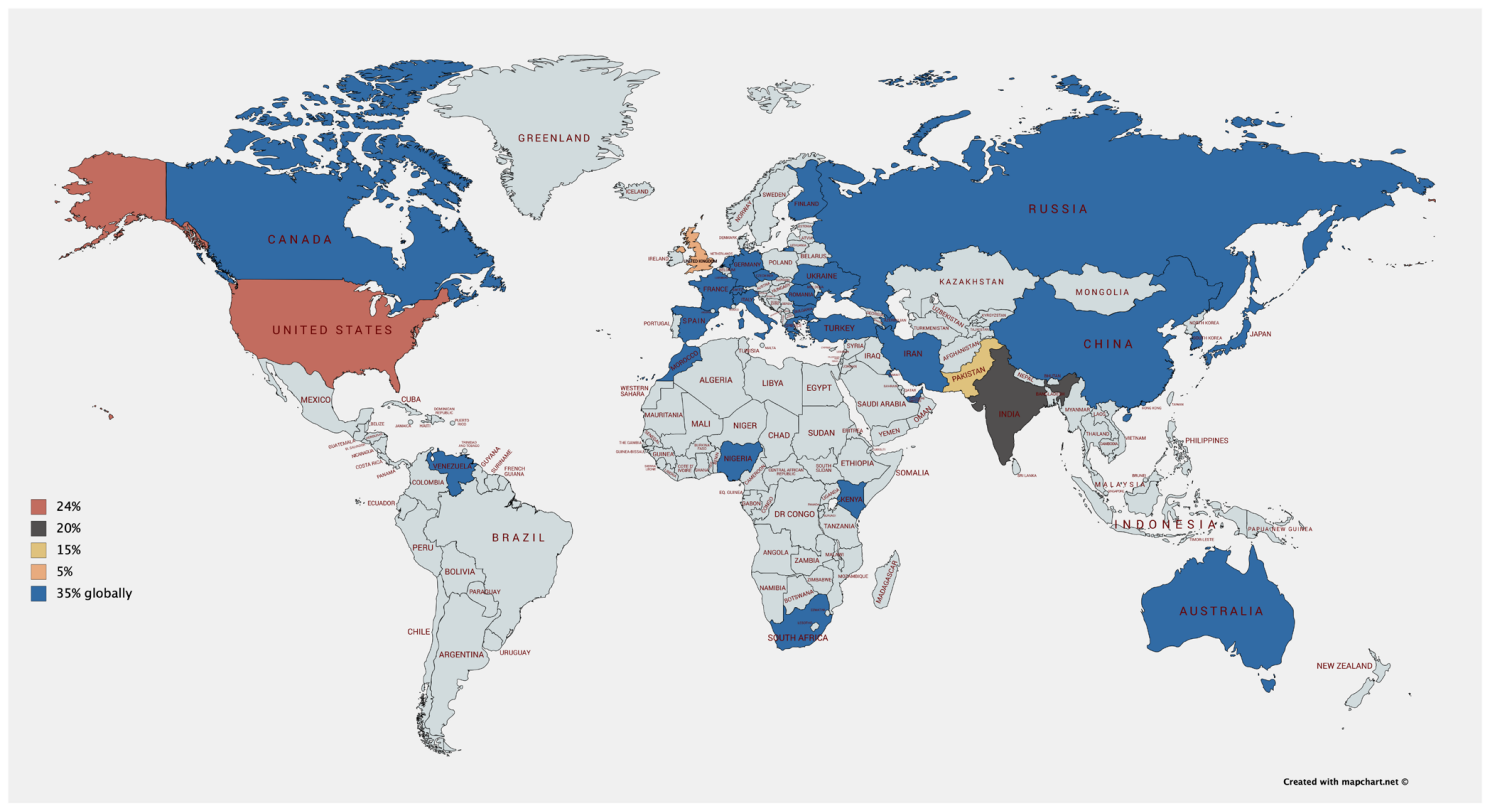
Distribution of journals discontinued from Scopus by country. The map chart shows the different frequencies of distribution by country with different colors.

Among the 135 publishers identified, those with the largest number of discontinued journals were: *Academic Journals Inc.* (39/317; 12.3%), *Asian Network for Scientific Information* (19/317; 6.0%), and *OMICS Publishing Group* (18/317; 5.7%). Table 1 reports the distribution of journals discontinued from Scopus by publisher. United States (76/317, 24%), India (63/317, 20%) and Pakistan (49/317, 15%) were countries most commonly declared as publisher headquarters ( Figure 1 and Table 2).

**Table 1.** Distribution of journals discontinued from Scopus by publisher.

Publishers (n=135)	% ( n )
Academic Journals Inc. Asian Network for Scientific Information OMICS Publishing Group Medwell Journals iMedPub World Scientific and Engineering Academy and Society Science Publications Academy Publisher Allied Academies Canadian Center of Science and Education International Digital Organization for Scientific Information (IDOSI) Science and Engineering Research Support Society Serials Publications (International Science Press) AMSE Press Eurojournals Inc Hikari Ltd Research India Publications Others	12.3 (39/317) 6 (19/317) 5.7 (18/317) 4.1 (13/317) 3.5 (11/317 2.8 (9/317) 2.5 (8/317) 2.2 (7/317) 1.9 (6/317) 1.9 (6/317) 1.9 (6/317) 1.6 (5/317) 1.6 (5/317) 1.3 (4/317) 1.3 (4/317) 1.3 (4/317) 1.3 (4/317) 47 (149/317)

Data are reported as percentages and fractions. Publishers with less than four journals discontinued from Scopus were grouped as ‘Others’.

**Table 2.** Distribution of journals discontinued from Scopus by country.

Country (n=33)	% ( n )
United States of America India Pakistan United Kingdom Turkey Greece Canada Finland France United Arab Emirates Italy Romania South Korea Ukraine Australia Bulgaria Others	24 (76/316) 19.9 (63/316) 15.5 (49/316) 5.4 (17/316) 4.1 (13/316) 3.5 (11/316) 3.2 (10/316) 2.5 (8/316) 2.2 (7/316) 1.9 (6/316) 1.6 (5/316) 1.6 (5/316) 1.6 (5/316) 1.6 (5/316) 1.3 (4/316) 1.3 (4/316) 8.8 (28/316)

Data were retrieved from Scimago Journal & Country Rank and are reported as percentages and fractions. Countries with less than four Scopus discontinued journals were grouped as ‘Others’

The subject areas with the greatest number of discontinued journals were *Medicine* (52/317; 16%), *Agriculture and Biological Science* (34/317; 11%), and *Pharmacology, Toxicology and Pharmaceutics* (31/317; 10%) Table 3 and Extended data Table 1 ^25^ report the distribution of discontinued journals by subject area and sub-area in full. Of these journals, 93% (294/317) declared they published using an Open Access model.

**Table 3.** Distribution of journals discontinued from Scopus by subject areas.

Medicine	16.4%	(52/317)
Agricultural and Biological Sciences	10.7%	(34/317)
Pharmacology, Toxicology and Pharmaceutics	9.8%	(31/317)
Engineering	7.9%	(25/317)
Computer Science	7.9%	(25/317)
Biochemistry, Genetics and Molecular Biology	5.4%	(18/317)
Business, Management and Accounting	5.4%	(17/317)
Mathematics	5.4%	(17/317)
Social Sciences	4.7%	(15/317)
Arts and Humanities	3.8%	(12/317)
Multidisciplinary	3.5%	(11/317)
Economics, Econometrics and Finance	2.5%	(8/317)
Environmental Science	2.2%	(7/317)
Immunology and Microbiology	2.2%	(7/317)
Materials Science	2.2%	(7/317)
Veterinary	2.2%	(7/317)
Earth and Planetary Sciences	1.6%	(5/317)
Chemistry	1.3%	(4/317)
Energy	1.3%	(4/317)
Chemical Engineering	0.9%	(3/317)
Physics and Astronomy	0.9%	(3/317)
Nursing	0.6%	(2/317)
Dentistry	0.3%	(1/317)
Health Professions	0.3%	(1/317)
Neuroscience	0.3%	(1/317)

Data were retrieved from Scopus and are reported as percentages and fraction

First subject area as displayed in Scopus. Note: a journal may have more than one subject area. Table 4 shows the characteristics and metrics of the journals at the time of their discontinuation.

The median time of Scopus coverage prior to discontinuation of the journals was 8 years (IQR 6–10, range 1–54). In total, 299 journals had been assigned to a SCImago quartile (Q); 39 of them (13%) listed in Q1 or Q2, and 260 in Q3 or Q4 (87%). Only ten of the discontinued journals had an Impact Factor at the year of discontinuation, with a median value of 0.84 (IQR 0.37–2.29, range 0.28–4).

**Table 4.** Journal characteristics at the year of Scopus discontinuation.

Scopus coverage (yrs.) ^*^	8 [6-10] (1-54)
**Time from Scopus discontinuation** (yrs.) ^*^	5 [4-6] (2-12)
**Impact Factor** ^†^	0.84 [0.37-2.29] (0.28-4)
**SjR** ^‡^	0.17 [0.13-0.23] (0.1-1.41)
**SNIP** ^§^	0.4 [0.23-0.65] (0-4.56)
**CiteScore ^°^**	0.32 [0.17-0.46] (0-10.33)
**SCImago Quartile**	
**Q1 (%, n)** **Q2 (%, n)** **Q3 (%, n)** **Q4 (%, n)**	3.3 (10/299) 9.7 (29/299) 40.8 (122/299) 46.1 (138/299)

Data are reported as medians, interquartile ranges [IQRs] and ranges (minimum value – maximum value) or as percentages and fractions. ^*^ No missing data. The analyses were conducted on all the 317 journals discontinued from Scopus. ^†^ Data were available and calculated for 10 journals. ^‡^ Data were available and calculated for 304 journals. ^§^ Data were available and calculated for 299 journals. ^°^ Data were available and calculated for 82 journals. SjR: SCImago Journal & Country Rank; SNIP: Source Normalized Impact *per* Paper; IF: Impact Factor

### Citation metrics

Table 5 shows the total number of documents and citations, the total number of documents per journal and the citations count before and after Scopus discontinuation. The total number of citations received after discontinuation was 607,261, with a median of 713 citations (IQR 254–2,056, range 0–19,468) per journal.

**Table 5.** Citations and documents before and after Scopus discontinuation.

**Total number of** **documents**	591968	
**Total number of citations**	1152779	
**Documents** ***per*** journal ^*^	429 [159.5–1244] (2–132482)	
	**Before Scopus** **discontintinuation**	**After Scopus** **discontintinuation**
**Citations (n)**	545518	607261
Citations *per* journal ^*^	415 [120-1580] (0-67529)	713 [254-2056] (0-19468)
Citations *per* year ^*^	51.75 [15.17- 144.3] (0- 2028)	152.9 [49.43-408] (0-4571)
Citations *per* document ^*^	1 [0.39-2.15] (0-17.12)	1.66 [0.93-2.66] (0-80.70)

Data are reported as medians, interquartile ranges [IQRs] and ranges (minimum value – maximum value) unless otherwise specified. * No missing data. Analyses were conducted on all the 317 journals discontinued from Scopus.

Paired *t*-tests (Wilcoxon matched-pairs signed rank test) revealed that the number of citations per year after discontinuation was significantly higher than before (median of difference 16.89 citations [-13.68-117.5] (-1427-3491), p<0.0001). Likewise, the number of citations per document proved significantly higher after discontinuation (median of difference 0.42 citations [-0.32-1.31] (-10.35-79.49), p<0.0001).

### Indexing in Cabell’s lists, updated Beall’s list, DOAJ and scientific databases

Among the discontinued journal, 22% (72/317) were included in the Cabell’s blacklist, while 29 (9%) were currently under review for inclusion. Only five journals (2%) were included in Cabell’s whitelist. In 243 cases (76.6%), either the journal publisher was included in the updated Beall’s list of predatory publishers or the journal was included in the corresponding list of standalone journals (76.6%). The DOAJ currently includes only 9 journals. In total, 61 journals were previously included and discontinued by DOAJ; in 36 cases the reason was ‘suspected editorial misconduct by the publisher’ in 23 instances it was ‘journal not adhering to best practice’ and in one case ‘no open access or license info’.

Table 6 shows the indexing in Web of Science, updated Beall’s list, Cabell’s white- and blacklist, and DOAJ (both included and discontinued) and the presence of articles in PubMed.

**Table 6.** Discontinued journals’ current Open Access policy and the indexing of their articles in major databases.

**Open Access journals (%, n)**	92.7 (294/317)
**PubMed (%, n)** *	6.3 (20/317)
**Web Of Science (%, n)**	9.1 (29/317)
**Beall’s List (%, n)**	76.6 (243/317)
**Cabell’s Whitelist (%, n)**	1.6 (5/317)
**DOAJ included (%, n)**	2.8 (9/317)
**Cabell’s Blacklist (%, n)**	22.7 (72/317)
**DOAJ discontinued (%, n)**	19.2 (61/317)

Data are reported as percentages and fractions. DOAJ: Directory of Open Access Journals * Proportion only of journals with at least one article indexed in PubMed (e.g. PubMed Central).

## Discussion

The present study aimed to scrutinize the main features of journals whose coverage was discontinued by Scopus due to publication concerns. To do so, (a) we counted and compared citation metrics per journal and per document obtained *before* and *after* discontinuation, and (b) we accessed established blacklists and whitelists dealing with the issue of predatory publishing, i.e. Cabell’s and updated Beall’s list, as well as the DOAJ.

Our main finding was that articles published in these journals before discontinuation remain available to users and continue to be cited after discontinuation, and even more so than before. Moreover, a large number of the discontinued journals are likely to be predatory.

A previous analysis conducted to evaluate the scientific impact of predatory publishing has concluded that “articles published in predatory journals have little scientific impact” ^26^. The study evaluated Google Scholar and Scopus citation statistics of 250 randomly sampled articles, that have been published in predatory journals in 2014. The citations were then compared to those of a control group of articles, published in journals included in Scopus database. Our study aimed to evaluate and describe the metrics and citations of all the journals discontinued from Scopus for ‘publication concerns’. At a secondary stage, the presence of these journals in the Cabells’ and Beall’s lists was investigated. The different purposes and designs of the two studies may explain the different findings.

Although Scopus rigorously controls content quality and warns users when a journal is discontinued in its source details, the average user rarely accesses journaldetails, usually focusing on article contents alone. As a result the reader remains unaware that the article they have accessed was issued by a journal discontinued for publication concerns. Therefore, articles issued by journals whose scientific reputation is currently deemed questionable continue to be cited as content from legitimate, up-to-standard journals. Quantification of the effect of discontinuation on the likelihood of citation shows that the articles published by these journals received significantly more citations after discontinuation than before.

**Figure 2.**
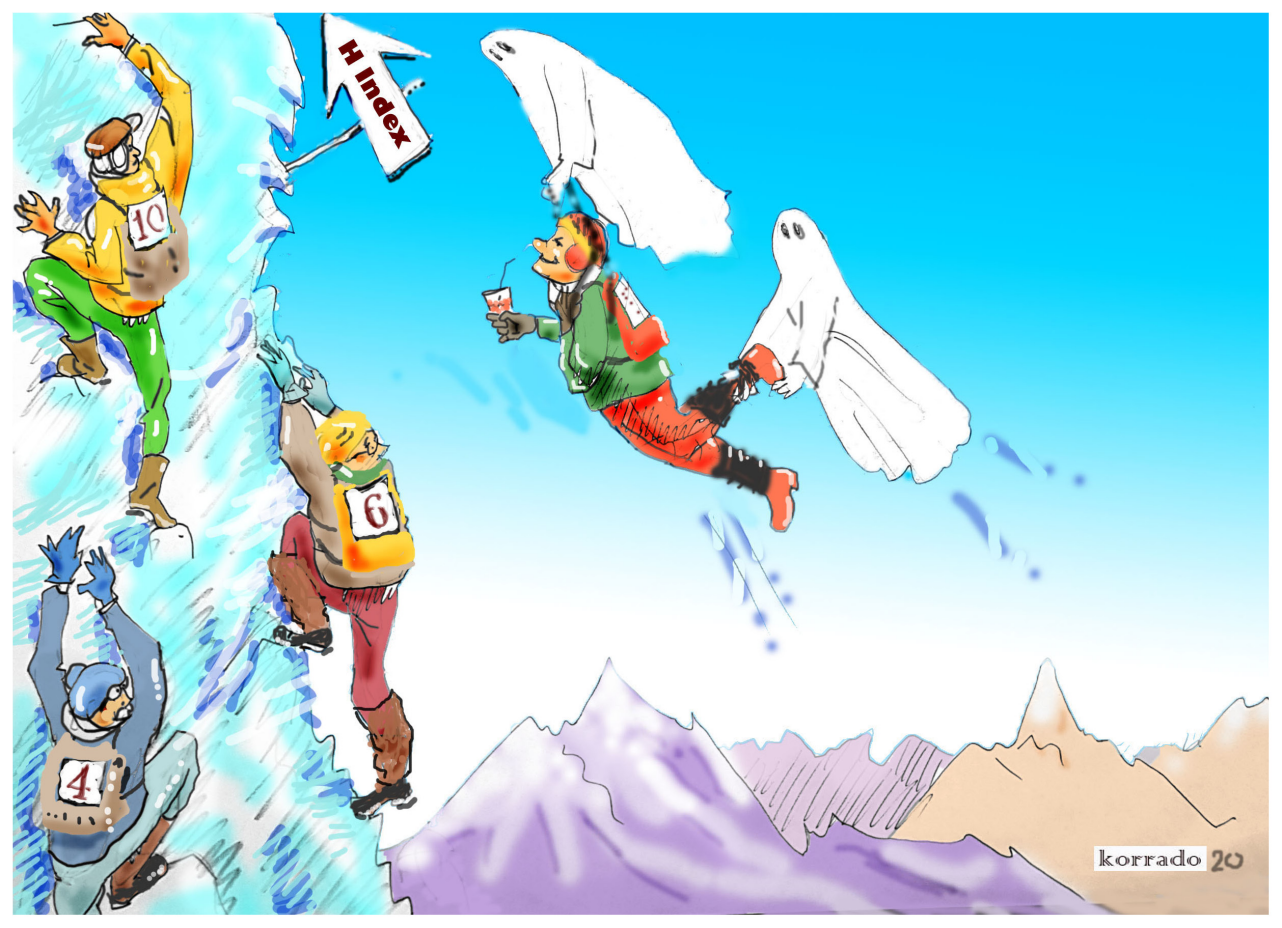
Discontinued journals may inflate authors’ metrics lifting them unnaturally and effortlessly.

Apart from dangerous exposure of scholars, clinicians and even patients to potentially dubious or low quality contents, citations from discontinued journals pose a serious threat to assessment of scientific merit and quality by institutions and academia. These citations contribute to the calculation of author metrics by Scopus. Among these metrics is included the Hirsch index (H-index) ^27^, a lead descriptor of productivity and scientific impact, upon which career advancements are often determined ^2– 4^. The fact that discontinued journals contribute to academic promotion is a pertinent issue, and has inspired the vignette depicted in Figure 2: discontinued journals may inflate authors’ metrics lifting them unnaturally and effortlessly.

Of greatest concern is our finding that many of the discontinued journals display predatory behaviors in claiming to be open access, without actually being indexed in DOAJ. Exploitation of the open-access publishing model has been shown to go hand in hand with deviation from best editorial and publication practices for self-interest ^7^. Predatory journals are not only associated with poor editorial quality, but are also deceptive and misleading by nature, i.e. they prioritize self-interest at the expense of scholars, and lack transparent and independent peer review ^9, 28^. Young researchers from low- and middle-income countries are probably most susceptible to the false promises and detrimental practices of predatory journals. However, “predatory scholars” also seem to exist, possibly sharing a common interest with deceptive journals and publishers, knowingly using them to achieve their own ends ^29, 30^.

The policy underlying the decision to keep publications prior to discontinuation of indexing is clear. Some of these publications may actually fulfill publishing criteria (e.g. International Committee of Medical Journal Editors, Committee on Publication Ethics). It would be unfair to punish researchers for an eventual deterioration in journal performance; changes in the standards employed by the journal may change over time and the researchers may be unaware of quality issues. On the other hand, as the integrity of the editorial process cannot be vouched for, it is ethically untenable to keep such data available without clearer warnings.

One measure that could be undertaken immediately is, for example, flagging of articles that have been published in discontinued journals with clearly visible information regarding journal discontinuation, its date and its cause. Submitting articles published a certain amount of time before journal discontinuation to post-publication open peer-review is also a possibility. However, as solutions to this problem must balance fairness towards publishing researchers with ensuring the correctness of the metrics and citations deriving from these journals, Scopus may need to to set criteria for deleting discontinued journals from the publicly available database or, in the least, stop tracking their citations. Such measures must only be applied by the CSAB case-by-case, after evaluating the full impact of such action and the severity of the potential misconducts. At the author-level, an alternative may be the provision of two metrics: one with and one without citations from publications in discontinued journals. 

This analysis is not free of limitations. First, this study lacks a control group of journals whose coverage had not been discontinued in the Scopus database. Therefore the differences we identified in the number of citations before and after discontinuation require further validation. Second, we included the year of discontinuation in the “ *after discontinuation*” period, starting from January 1 ^st^. This decision may have led to some overestimation in the number of citations received after discontinuation. Third, we included only those journals discontinued from Scopus for “publication concerns” but were not able to retrieve details regarding the specific concern raised. Finally, we did not evaluate the impact of the citations received after discontinuation on author-level metrics.

## Conclusions

Journals whose coverage in Scopus has been halted for publication concerns continue to be cited. This paradox may influence scholar metrics, potentially prompting career advancements and promotions. Further studies are needed, also investigating the journals discontinued from Scopus using the criteria “outlier performance – radar”, particularly effective in flagging potential predatory journals. Countermeasures should be taken to ensure the validity and reliability of Scopus metrics for both journals and authors due to their importance for scientific assessment of scholarly publishing. Creative thinking is required to resolve this issue without punishing authors who have inadvertently published good quality papers in a failing or predatory discontinued journal.

## Data availability

### Source data

Discontinued sources from Scopus are available from the following link: https://www.elsevier.com/__data/assets/excel_doc/0005/877523/Discontinued-sources-from-Scopus.xlsx

 All the relevant data are freely retrievable from Scopus ‘journal overview’ or can be requested to Scopus through https://www.scopus.com/sources.

### Underlying data

Figshare: Underlying data Table 1.xlsx. https://doi.org/10.6084/m9.figshare.12231083.v3 ^11^

This project contains the following underlying data:

- Underlying data Table 1.xlsx (Standardized data extraction form with data collected)

### Extended data

Figshare: Extended data Appendix 1.

https://doi.org/10.6084/m9.figshare.12231110.v2 ^24^

This project contains the following extended data:

- Extended data Appendix 1.docx (Definitions of sources and metrics used in the manuscript of the GhoS(t)copus Project)

Figshare: Extended data Table 1. https://doi.org/10.6084/m9.figshare.12233171.v2 ^25^

This project contains the following extended data:

- Extended data table.docx (Distribution of Scopus discontinued journals by subject sub-areas)

Data are available under the terms of the Creative Commons Attribution 4.0 International license (CC-BY 4.0).

## References

[1] Elsevier: How Scopus works. [accessed 28 February 2020]. Reference Source

[2] Cortegiani A Manca A Lalu M: Inclusion of predatory journals in Scopus is inflating scholars’ metrics and advancing careers. *Int J Public Health.* 2020;65:3–4. 10.1007/s00038-019-01318-w 31822951

[3] Bagues M Sylos-Labini M Zinovyeva N: A walk on the wild side: “Predatory” journals and information asymmetries in scientific evaluations. *Research Policy.* 2019;48(2):462–477. 10.1016/j.respol.2018.04.013

[4] Hedding DW: Payouts push professors towards predatory journals. *Nature.* 2019;565(7739):267 10.1038/d41586-019-00120-1 30644448

[5] Holland K Brimblecombe P Meester W: The importance of high-quality content: curation and re-evaluation in Scopus. [accessed 28 February 2020]. Reference Source

[6] Elsevier: Content Policy and Selection. [accessed 28 February 2020]. Reference Source

[7] Grudniewicz A Moher D Cobey KD: Predatory journals: no definition, no defence. *Nature.* 2019;576(7786):210–212. 10.1038/d41586-019-03759-y 31827288

[8] Cortegiani A Longhini F Sanfilippo F: Predatory Open-Access Publishing in Anesthesiology. * Anesth Analg.* 2019;128(1):182–187. 10.1213/ANE.0000000000003803 30234529

[9] Severin A Low N: Readers beware! Predatory journals are infiltrating citation databases. *Int J Public Health.* 2019;64(8):1123–1124. 10.1007/s00038-019-01284-3 31342093

[10] Elsevier: Scopus®, registered trademark of Elsevier B.V. [accessed 28 February 2020]. Reference Source

[11] Cortegiani A Ippolito M Ingoglia G: Underlying data Table 1: Standardized data extraction form with data collected. 2020. 10.6084/m9.figshare.12231083.v3

[12] Scimago Lab, Copyright 2007-2020. [accessed 28 February 2020]. Reference Source

[13] Journal Citation Reports. Copyright 2020. [accessed 28 Februrary 2020]. Reference Source

[14] Centre for Science and Technology Studies: About CWTS.Leiden University, The Netherlands. [accessed 28 February 2020]. Reference Source

[15] Beall’s list of predatory journals and publishers. [accessed 28 February 2020]. Reference Source

[16] Directory of Open Access Journals. Licensed under CC BY-SA. [accessed 28 February 2020]. Reference Source

[17] PubMed Help. Bethesda (MD): National Center for Biotechnology Information (US);2005; PubMed Help. [Updated 2019 Jul 25]. [accessed 28 February 2020]. Reference Source

[18] Clarivate Analytics Company. [accessed 28 February 2020]. Reference Source

[19] Cabell’s Scholarly Analytics. [accessed 28 February 2020]. Reference Source

[20] Directory of Open Access Journals: DOAJ publishes lists of journals removed and added. Directory of Open Access Journals Blog. [accessed 28 February 2020]. Reference Source

[21] Strinzel M Severin A Milzow K: Blacklists and Whitelists To Tackle Predatory Publishing: a Cross-Sectional Comparison and Thematic Analysis.Wolf JM, editor. *mBio.* 2019;10(3): pii: e00411-19. 10.1128/mBio.00411-19 31164459 PMC6550518

[22] Winkler WE: String Comparator Metrics and Enhanced Decision Rules in the Fellegi-Sunter Model of Record Linkage.ERIC.1990 Reference Source

[23] Porter EH Winkler WE: Approximate string comparison and its effect on an advanced record linkage system.Citeseer.1997 Reference Source

[24] Cortegiani A Ippolito M Ingoglia G: Extended data Appendix 1. *figshare.*Online resource.2020 10.6084/m9.figshare.12231110.v2

[25] Cortegiani A Cugusi L Ippolito M: Extended data Table 1. *figshare.*Dataset.2020 10.6084/m9.figshare.12233171.v2

[26] Bjork BC Kanto-Karvonen S Harviainen JT: How Frequently Are Articles in Predatory Open Access Journals Cited. * Publications.* 2020;8(2):17 10.3390/publications8020017

[27] Hirsch JE: An index to quantify an individual’s scientific research output. *Proc Natl Acad Sci U S A.* 2015;102(46):16569–16572. 10.1073/pnas.0507655102 16275915 PMC1283832

[28] Butterworth JF 4th Vetter TR: Predatory Journals Undermine Peer Review and Cheapen Scholarship. *Anesth Analg.* 2019;128(1):11–12. 10.1213/ANE.0000000000003862 30550469

[29] Cobey KD Grudniewicz A Lalu MM: Knowledge and motivations of researchers publishing in presumed predatory journals: a survey. *BMJ Open.* 2019;9(3):e026516. 10.1136/bmjopen-2018-026516 30904874 PMC6475169

[30] Pond BB Brown SD Stewart DW: Faculty Applicants' Attempt to Inflate CVs Using Predatory Journals. *Am J Pharm Educ.* 2019;83(1):7210. 30894776 10.5688/ajpe7210 PMC6418842

