# Supplementary material for: Citations and metrics of journals discontinued from Scopus for publication concerns: the GhoS(t)copus Project
Source: F1000Res. 2020 Aug 26;9:415. Originally published 2020 May 21. [Version 2] doi: 10.12688/f1000research.23847.2 (PMC7512033; doi:10.12688/f1000research.23847.2)
Supplement: Peer review history

# Peer review: Reviewer response for version 2

**DOI:** 10.5256/f1000research.28667.r71207

**Published:** 2020-09-23

**Authors:** Arash Afshari

**Affiliations:** 1 Department of Pediatric and Obstetric Anesthesia, Rigshospitalet, Juliane Marie Center, University of Copenhagen, Copenhagen, Denmark

I like to thank the authors and the journal for considering me as a peer for this review. I very much enjoyed this revised paper and certainly the indebt revision of the comments from the first round. I only have few general comments on the external validity of this paper:

 Exclusion of journals only based on publication concerns may not reflect the complexity facing us of information overload.

 The proxy control group, which is absent and is mentioned by the authors is in reality a comparison with journals using an editorial system based on peer review as the gold standard that ensures adequate quality. But considering the issues surrounding the famous hydroxychloroquine paper in Lancet and the change of their editorial policy this week (not published in detail how this will affect the peer review system), the complexity of this issue becomes even more apparent.

 I personally am far from convinced that the current editorial system and peer review is at all adequate or up-to-date to detect scientific fraud and ensure high quality papers. What we see is an inflation in the number of journals opting for a payment system. Thus, the financial incentives for publication and the consequent pressure on editors is increasing. This is not really addressed in this paper. Additionally, the journals are in essence cherry picking. Peer reviewers do the job, researchers do their part and the funding comes from public or private sources. The journals end up making the money. And there is often very little transparency about the quality of editorial system and the peer review process in even very well-established papers. For instance, one never knows the number of peers, name, affiliation, conflict of interest and the extent of data scrutiny. Finally, often data are not provided, shared and even to a lesser degree re-analyzed unless very controversial or with a high clinical impact. And there is often very little effort nor incentive to opt for reproducibility.

 What we are witnessed to is a tsunami of useless scientific papers. For instance only 3 percent of systematic reviews published today have adequate quality and address the issue of random error and reproducibility. Thus, a major limitation of this study is that it only reflects the general quality of journals as a proxy indicator for scientific malpractice and retraction per se.

 But overall, an enlightening work that adds valuable information to complexity of the issue of predatory journals and their impact.

Is the work clearly and accurately presented and does it cite the current literature?

Yes

If applicable, is the statistical analysis and its interpretation appropriate?

Yes

Are all the source data underlying the results available to ensure full reproducibility?

Yes

Is the study design appropriate and is the work technically sound?

Yes

Are the conclusions drawn adequately supported by the results?

Yes

Are sufficient details of methods and analysis provided to allow replication by others?

Yes

Reviewer Expertise:

EBM, anaesthesia, critical care

I confirm that I have read this submission and believe that I have an appropriate level of expertise to confirm that it is of an acceptable scientific standard.

# Peer review: Reviewer response for version 1

**DOI:** 10.5256/f1000research.26314.r64881

**Published:** 2020-07-31

**Authors:** Nadia Elia, Pablo Iriarte, Floriane Muller

**Affiliations:** 1 Library of the University of Geneva, Geneva, Switzerland; 2 Division of Anaesthesiology, Department of Acute Medicine, Geneva University Hospitals, Institute of Global Health, Faculty of Medicine, University of Geneva, Geneva, Switzerland

Thank you for giving us the opportunity to read this article in which the authors describe the characteristics, citations and metrics of journals that have been indexed in the Scopus database, at some point, and have afterwards been “discontinued” within Scopus for different reasons, summarised as “publication concerns”.

 Since the articles that have been published before the journal’s indexation was discontinued remain in the database, and can still be found, they may still be cited.

 The authors find this to be particularly problematic since they believe that these journals may be what are often called “predatory journals”, and therefore may threaten the credibility of science, by polluting the database with “weak research”.

 Therefore, the authors aimed to compare the number of citations per year, and per journal, and per document, before and after the journal was delisted. They conclude that the number of citations was actually higher after the journal was discontinued from Scopus.

 Although we understand the problem these authors try to highlight, we have some major concerns regarding some aspects of this study (mainly related to baseline assumptions and lack of clear definition) and also some minor ones.

**Major concerns:**

**Baseline assumption: **In this article, the authors suggest that if a journal is being indexed, even for a long period of time (half of them have been indexed for 8 to 54 years), and is encountering “publication concern”, then all the previously published articles should become suspicious of bad science. We are not sure this should be considered straight forward, for the reason developed under our second major concern.

**“Predatory journals”:** the problem of the lack of a clear definition of what a predatory journal is, remains. The authors use different sources to try to identify journals as “predatory” and we can only realise that the sources do not seem to agree. Although authors auto cite their own “consensus definition” of predatory journals and publishers “(..) entities that prioritize self-interest at the expense of scholarship and are characterized by false or misleading information, deviation from best editorial and publication practices, a lack of transparency, and/or the use of aggressive and indiscriminate solicitation practices.” they fail to underline that not everybody agrees with this definition. Also, the recent COVID-19 debacle of very low-quality scientific publications, published in usually highly regarded journals, suggests that bad peer-review and misleading articles may not be a characteristic of any journal.

 Also, it remains unclear to us how a journal may be indexed for 8 to 10 years, and all of a sudden become “predatory”. Or was it predatory in the first place, but was only uncovered after such a long time? If this is what the authors suggest, then what should we think about “recently” indexed journals? They may all be predatory as well, and will only be uncovered in 5 to 10 years?

**“Publication concerns”:** This term needs to be better defined in order to really understand what lies behind it. It remains unclear why these journals have been excluded from the Scopus database at some point. Interestingly, half of these journals have been deemed good enough to figure in the database for more than 8 years… that’s a lot! And all of a sudden, they are not judged acceptable anymore and are discontinued from Scopus. Ok, why not. It may take some time before someone alerts Scopus of the misbehaviour of a given journal, although more than 10 years for 25% of them seems a lot. Or could it be a problem behind the vague concept of “publication concerns”? Could it be that the publication has stopped? Or the journal has changed its name? Or has merged with another one? Or has changed in quality over time? Illustrating some of the reason for discontinuation would help the reader understand the context.

 According to Scopus’ document cited in the article (ref. 5), there are 3 causes prompting Scopus to launch a journal re-evaluation: Under performance – metrics ; Outlier performance – radar ; Publication concerns. It might have been interesting to analyse the journals removed using the criteria “outlier performance – radar” as well as, according to Scopus document (ref. 5 cited by the authors) it is “particularly effective in flagging potential predatory journals.” Scopus describes it as ”an algorithm that flags journals based on approximately 40 outlier predictors, including sudden change in output volume, sudden change in publishing country and/or affiliations, and high journal/author self-citation rates.”

**Increase in citations:** The authors are worried that the citations of these journal have increased after the journal’s indexation was discontinued in the Scopus database. The problem here is that they do not seems to consider the fact that this may be the case for all journals (those indexed and those discontinued) which is probably due to the rapid increase in the number of publications over time. Unfortunately, this study lacks a “control group” (journals whose coverage has NOT been discontinued in the Scopus database) which could have help the reader understand whether the increase in citation of these journals was similar, was higher, or was lower than that of “legitimate journals”.

**Underlying discourse**: The term "inflated" used in the title, in Figure 2 and conclusion suggests manipulation or distortion of citations and an artificial advantage for authors of articles published in predatory journals before they are removed from Scopus. This is not demonstrated by the reasoning and data used in the article as a basis for comparison is missing.

**Methods and reproducibility: **While the authors have provided data alongside the article, we have not been able to reproduce some of their results, such as “citations per year” presented in table 5. Data presented in “underlying data table 1” would benefit from better variable descriptions, such as where exactly was the information collected from, and the date of its collection.

 Some variable names and analysis are misleading, such as “Actual Pubmed”, described in methods section as “inclusion in PubMed” and in table 6 as “main database indexing”. It does not reflect whether the journal is currently indexed in PubMed, but may in some cases only indicate that a single article is present in PubMed or selected citations, due to their deposit in PMC (eg. “Advanced Materials Letters“).

 Some data seem a bit bizarre… and information provided by the authors like “Citation before and after the date of discontinuation were manually counted based on either the Scopus journal overview or the downloadable tables made available by Scopus upon request (see source data)” (p.3) did not allow us to double check some numbers that were weirdly extreme, and potential typos. Some counts of the number of citations seem erroneous, leading to an aberrant number of citations per document for journals like "Mental Health in Family Medicine" (80 citations per document before discontinuation) or "Pharmacognosy Reviews" (170 after).

 Other example of bizarre data: according to “underlying data table 1” the journal “Advanced material research” has been indexed for 10 years (from 2004 to 2014) and has received during this period only 3 citations. However, after having been delisted from the Scopus database, during a 6 year period (2014 to 2019), it has received 13875 citations. Any thoughts on how/why this could have happened?

**Minor concerns:**

**Abstract**:

- Background: “contains the largest number of abstract and articles…” -> "One of the largest" could be better, some databases are bigger than Scopus (Google Scholar and Dimensions, see https://doi.org/10.1016/j.joi.2018.03.006 ^1^ or https://arxiv.org/abs/2004.14329)
- Background: The term “publication concerns” may not be clear to everyone.
- Background: “These journals remain indexed and can be cited.” This sentence is confusing. The articles published before the exclusion remains indexed and their citations continue to be taken into account but new issues of the journals are not indexed any more.
- Methods: The use of the term “discontinued” both for DOAJ (Results) and for journal publication (Background) is confusing. Should we say “excluded” or “delisted”?
- Results: “317 journals were evaluated” but next sentence states ninety-three percent of the journals (294/318)” -> typo for 318?
- Results: “the mean number of citations per year after discontinuation was significantly higher than before, and so was the number of citation per document”. Unclear whether the median difference of 64 is per journal, or cumulative across all “discontinued” journals? What are “documents”? Do you mean “articles”? or are there any other types of publication?
- Conclusions: it’s unclear how the conclusion regarding “predatory journals” is drawn. Also, we don’t think the career advancement are “artificial”, they are real! Although maybe “undue”?

**Introduction**:

-  “publications from no longer indexed journals may not be removed retrospectively … hence articles … could remain part of the database ^7^” p.3 -> this conditional statement seems to contradict abstract which categorically states “These journals remains indexed” as well as the author’s conclusion “we propose that CSAB could apply these measure case-by-case”. Reference 7, linked with statement, was not helpful to clarify.    

**Methods**:

- “Independently collected by eight of the authors in pairs”: not very clear: two by two, or checked by two different people independently?
- “the year of our data collection”: more precision maybe?

**Results**:

- Why were data from 31 journals not retrieved? What was the problem?
- Table 1: Interestingly, none of these journals (discontinued from Scopus for publication concerns) have been published by Elsevier. Could there possibly be a selection bias? An interesting option could be to check if Elsevier’s journals that are still in Scopus might have been discontinued from others sources (DOAJ, WOS), and on which grounds?
- Table 1: Maybe the table could be enhanced to provide information about whether other journals by that publisher are still included in Scopus or not? Eg. 39 journals discontinued from Scopus were published by Academic journal Inc. Are there any other journals by this publisher still in Scopus?
- Table 3: don’t need 2 decimal precision in %.
- Table 3: Subject area are repetitive in Scopus, and a journal can have more than one, while this table and underlying data mention only 1 per journal, presumably the first appearing in Scopus ? If so, probably worth indicating and better to remove the percentages in table 3 falsely suggesting mutually exclusive categories?
- Table 5 and results (text): unclear where the median difference of 64 comes from? Or the 0.4?
- Table 5: total number of citation does not match number of citation before and after discontinuation. Any thoughts on how/why this could have happened? A note of explanation about that would be useful.
- Citations by year in table 5: The number of years before and after the journal is removed from Scopus is very different, the average is more than 9 years before and 4 years after (median of 8 and 4 respectively) which makes the comparison in Table 5 not relevant. Indeed, the number of citations per year is higher during the 2 or 3 years following the publication of the article and decreases sharply with time (DOI:10.1371/journal.pone.0153730 ^2^) so that the ratio of citations per year also decreases if a larger number of years is used.
- Distribution of articles: of the 317 journals analysed, 5 contain more than half of the articles concerned by this question. This very inhomogeneous distribution means that the statistical analyses and the percentages per journal do not take this kind of distribution into account.
- Page 5, first paragraph: table 2 should probably read table 5?
- Page 5, 2 ^nd^ paragraph: In 243 case (243/317)… is useless here. Maybe the authors meant 76.6% (243/217)
- Table 6: maybe a good idea to separate the “positive” facts (being indexed in Pubmed, WoS or Cabell’s whitelist) from the negative ones (Beall’s list, Cabell’s blacklist DOAJ discontinued.)

**Discussion**:

- p.7 Unclear why the term “ghost journal” suddenly appear and how it is defined.
- p. 7-8 “Of greatest concern is our finding that many of the discontinued journals display predatory behaviors in claiming to be open access” -> do you mean “displaying… are claiming”? to our understanding the article does not say that open access systematically means predatory. According to ref 9 et 22, the large majority of DOAJ indexed journals were not found in Beall’s list or Cabell’s Blacklist.
- p. 8: “Such journals” unclear: predatory journals or OA journals?
- The authors highlight that a limitation of their methodology is that they have included the year of discontinuation in the period “after discontinuation”, which could have led to overestimations. Then why not present the 2 analyses with the year of discontinuation included in the period BEFORE and in the period AFTER discontinuation, so that the reader can check for him-herself what bias this has induced?
- A mention of or comparison with other databases’ practices with regards to removing journals for indexing could be interesting. Do their approaches differ from Scopus’?

**Conclusions**:

- Proposals are missing to solve the problem addressed and to avoid the stigmatization of the authors of the "suspect" articles. For example, a new open peer-review for articles published within X months before the journal's exclusion would be a possibility.
- Maybe another idea would be to flag published articles that have been published in journals that are not indexed anymore “NB: this article was been published in YEAR, in a journal that has encountered publication concerns in YEAR”

**References**:

- Reference 5 URL should be https://www.elsevier.com/__data/assets/pdf_file/0004/891058/ACAD_LIB_SC_ART_Importance-of-high-quality-content_WEB.pdf
- Link in reference 12 does not work properly due to a superfluous space in the middle.
- Reference style is not harmonized (cf. 1 ^st^ § of methods section makes 4 references to the same underlying data table 1 (ref number 12 in the reference list, 2 of whom are not correct and should refer to source data, the rest having various citation style).
- There might be some mix up in references: ex. §4 on page 3 lists ref 6-8. Is it possible it should read 5, 7-8 instead?
- The literature review would benefit from additional references to complete or contrast with the author’s findings: ex. doi:10.3390/publications8020017 ^3^ that concludes that “articles published in predatory journals have little scientific impact.”
- Auto-citations: There are different ways to increase a researchers’ number of citations or H-index. Publishing in a “predatory journal” may be one of them, but auto-citation is also one. Of the 30 references cited at the end of this paper, 11 (37%) are auto-citations (citation of a reference including at least on author of the present paper), 7 (23%) are articles from others, and the remaining 12 were websites.

**Typo/language**

- In Underlying data table 1: last column title - DOAJ instead of DOAH ?
- p.3 §3 “journal are no longer be indexed” -> “journal are no longer indexed”
- p.3 §5 “still they can get” -> “they can still get”

Reviewer Expertise:

NA

We confirm that we have read this submission and believe that we have an appropriate level of expertise to confirm that it is of an acceptable scientific standard, however we have significant reservations, as outlined above.

## References

[1] : Dimensions: A competitor to Scopus and the Web of Science?. *Journal of Informetrics*.2018;12(2) : 10.1016/j.joi.2018.03.006 430-435 10.1016/j.joi.2018.03.006

[2] : From Excessive Journal Self-Cites to Citation Stacking: Analysis of Journal Self-Citation Kinetics in Search for Journals, Which Boost Their Scientometric Indicators. *PLoS One*.2016;11(4) : 10.1371/journal.pone.0153730 e0153730 10.1371/journal.pone.0153730 27088862PMC4835057

[3] : How Frequently Are Articles in Predatory Open Access Journals Cited. *Publications*.2020;8(2) : 10.3390/publications8020017 10.3390/publications8020017

# Response

**Published:** 2020-08-13

**Authors:** Andrea Cortegiani

**Affiliations:** University of Palermo, Italy

Dear reviewers,

 We are glad to submit a revised version of our manuscript, previously entitled ‘ **Inflated citations and metrics of journals discontinued from Scopus for publication concerns: the GhoS(t)copus Project** **’**. The comments you provided were more than helpful in revising and improving the manuscript.

 We hereby provide a point-by-point reply to the comments.

 Best regards,

 Andrea Cortegiani, MD

 On behalf of co-authors

 _______________________________________________________________________

**Response to Reviewers team 2** (Pablo Iriarte, Library of the University of Geneva, Geneva, Switzerland; Floriane Muller, Library of the University of Geneva, Geneva, Switzerland; Nadia Elia, Division of Anaesthesiology, Department of Acute Medicine, Geneva University Hospitals, Institute of Global Health, Faculty of Medicine, University of Geneva, Geneva, Switzerland)

**Comment 1**: *Thank you for giving us the opportunity to read this article in which the authors describe the characteristics, citations and metrics of journals that have been indexed in the Scopus database, at some point, and have afterwards been “discontinued” within Scopus for different reasons, summarised as “publication concerns”.*

*Since the articles that have been published before the journal’s indexation was discontinued remain in the database, and can still be found, they may still be cited.*

*The authors find this to be particularly problematic since they believe that these journals may be what are often called “predatory journals”, and therefore may threaten the credibility of science, by polluting the database with “weak research”.*

*Therefore, the authors aimed to compare the number of citations per year, and per journal, and per document, before and after the journal was delisted. They conclude that the number of citations was actually higher after the journal was discontinued from Scopus.*

*Although we understand the problem these authors try to highlight, we have some major concerns regarding some aspects of this study (mainly related to baseline assumptions and lack of clear definition) and also some minor ones.*

**Reply**: We are very grateful for the insights of the reviewers which have led us to improve our manuscript. We have now submitted a revised version of the manuscript and herein is our point-by-point reply to the comments. English form was also revised.

 Major comments: 

**Comment 2:** *Baseline assumption: In this article, the authors suggest that if a journal is being indexed, even for a long period of time (half of them have been indexed for 8 to 54 years), and is encountering “publication concern”, then all the previously published articles should become suspicious of bad science. We are not sure this should be considered straight forward, for the reason developed under our second major concern.*

**Reply:** Thank you for the opportunity to clarify this. We made no such claim in the paper and, in fact, our key message was different. We clearly stated in the discussion that “It would be unfair to punish researchers for an eventual deterioration in journal performance; changes in the standards employed by the journal may change over time and the researchers may be unaware of quality issues”. We aimed to provide an analysis and describe the main scientific features and citation metrics of journals discontinued from Scopus for publication concerns as we strongly believe that this phenomenon merits discussion. We fully agree with the reviewer that further evaluation is required before the best solution for all aspects of this complex issue is determined. In fact, this study is the first to provide some of the information required to answer this question, albeit not all. We also agree with the reviewer regardless of the solution that is decided upon in the future, it should ensure that researchers are not unfairly punished. This is clearly stated. However, as we also point out, this issue can no longer be ignored; it involves a large number of journals and published documents.

**Comment 3:** *“Predatory journals”: the problem of the lack of a clear definition of what a predatory journal is, remains. The authors use different sources to try to identify journals as “predatory” and we can only realise that the sources do not seem to agree. Although authors auto cite their own “consensus definition” of predatory journals and publishers “(..) entities that prioritize self-interest at the expense of scholarship and are characterized by false or misleading information, deviation from best editorial and publication practices, a lack of transparency, and/or the use of aggressive and indiscriminate solicitation practices.” they fail to underline that not everybody agrees with this definition. Also, the recent COVID-19 debacle of very low-quality scientific publications, published in usually highly regarded journals, suggests that bad peer-review and misleading articles may not be a characteristic of any journal.*

**Reply**: First, we would like to highlight that the definition we reported for “predatory” is not the authors’ own. It was taken from an international collaboration of 35 authors who extensively studied the topic. Although we agree that no definition is perfect, this is most certainly not something we decided on ourselves and a consensus process was involved in its determination. If the reviewer wishes to argue with the definition provided, this should ideally be taken up with those involved in the consensus process. We surveyed recognized lists (i.e. Cabell, updated Beall, DOAJ) to evaluate the extent of predatory journals among the discontinued journals.

 With regards to the comment regarding the quality of COVID-19 research: Very true. We too have been following this topic with great interest. However, two wrongs do not make a right. In fact, this precise issue makes the discussion of journal metrics and our responsibilities towards them even more pertinent. Our research highlights some of the issues that arose with monitoring of the publication process from a different angle. It also promotes the need to continue to increase awareness within the scientific community itself regarding the damage that could potentially be caused by low-quality papers. 

**Comment 3:** *Also, it remains unclear to us how a journal may be indexed for 8 to 10 years, and all of a sudden become “predatory”. Or was it predatory in the first place, but was only uncovered after such a long time? If this is what the authors suggest, then what should we think about “recently” indexed journals? They may all be predatory as well, and will only be uncovered in 5 to 10 years?*

**Reply**: It is our impression that the process may occur in two manners: (1) Some of the more recently indexed journals may indeed turn out to be predatory. So indeed perhaps newly indexed journals need to undergo more rigorous monitoring than well established journals. Whether our impression is correct and, if so, how this should be done, are questions far beyond the scope of our research; (2) Some of the discontinued older journals probably did deteriorate slowly. Our impression was that this process is typically a “slippery slope” and does not have an abrupt cutoff. As our analysis was not intended to study this question, we prefer not to speculate on the ideal timing for journal discontinuation. More data and expert input is needed on how to identify this process in the future.

**Comment 4:** *“Publication concerns”: This term needs to be better defined in order to really understand what lies behind it. It remains unclear why these journals have been excluded from the Scopus database at some point. Interestingly, half of these journals have been deemed good enough to figure in the database for more than 8 years… that’s a lot! And all of a sudden, they are not judged acceptable anymore and are discontinued from Scopus. Ok, why not. It may take some time before someone alerts Scopus of the misbehaviour of a given journal, although more than 10 years for 25% of them seems a lot. Or could it be a problem behind the vague concept of “publication concerns”? Could it be that the publication has stopped? Or the journal has changed its name? Or has merged with another one? Or has changed in quality over time? Illustrating some of the reason for discontinuation would help the reader understand the context.*

**Reply**: The term ‘publication concerns’ is not one which spontaneously decided upon. It is the term defined and used by Scopus. Indeed, we report in the manuscript all the available definitions and details provided by Scopus. Unfortunately, no additional details are publicly available regarding the criteria used to discontinue a journal because of ‘publication concerns’. We too would be delighted to receive more details as they may be important. Having said this, we honestly doubt that merging with another paper or changing a journal name is cause for publication concern.  

 With regards to the reviewers’ rumination on the time gap for discontinuation: As noted above, it is indeed possible that some journals have changed quality over time or that they were evaluated only several years after indexing. This information would most certainly be interesting if it were publicly available, but it is not. Furthermore, as also stated above, this is not within the scope of our project.

**Comment 5:** *According to Scopus’ document cited in the article (ref. 5), there are 3 causes prompting Scopus to launch a journal re-evaluation: Under performance – metrics ; Outlier performance – radar ; Publication concerns. It might have been interesting to analyse the journals removed using the criteria “outlier performance – radar” as well as, according to Scopus document (ref. 5 cited by the authors) it is “particularly effective in flagging potential predatory journals.” Scopus describes it as ”an algorithm that flags journals based on approximately 40 outlier predictors, including sudden change in output volume, sudden change in publishing country and/or affiliations, and high journal/author self-citation rates.*

**Reply: **We thank the reviewer for this insightful comment which suggests some new research directions for the future. We have added this suggestion to the concluding paragraph of our paper. 

**Comment 6:** *Increase in citations: The authors are worried that the citations of these journal have increased after the journal’s indexation was discontinued in the Scopus database. The problem here is that they do not seems to consider the fact that this may be the case for all journals (those indexed and those discontinued) which is probably due to the rapid increase in the number of publications over time. Unfortunately, this study lacks a “control group” (journals whose coverage has NOT been discontinued in the Scopus database) which could have help the reader understand whether the increase in citation of these journals was similar, was higher, or was lower than that of “legitimate journals”.*

**Reply:** Please see below our response to this and the next comment together. 

**Comment 7:** *Underlying discourse: The term "inflated" used in the title, in Figure 2 and conclusion suggests manipulation or distortion of citations and an artificial advantage for authors of articles published in predatory journals before they are removed from Scopus. This is not demonstrated by the reasoning and data used in the article as a basis for comparison is missing.*

**Reply: **Indeed, the lack of a control group is a study limitation. We now point this out in the discussion section (see page 13). However, the authors have no interest vested in presenting an “underlying discourse” we have taken this comment very seriously. We have now removed the term “inflated” from both the title and the conclusions. We also modified the caption of Figure 2, substituting ‘can’ with ‘may’.

**Comment 8.1:** *Methods and reproducibility: While the authors have provided data alongside the article, we have not been able to reproduce some of their results, such as “citations per year” presented in table 5. Data presented in “underlying data table 1” would benefit from better variable descriptions, such as where exactly was the information collected from, and the date of its collection.*

**Reply: **We salute the reviewer for being so thorough as to attempt to reproduce our results.  

 Our decision to submit the full database for publication and to select an Open Research publishing platform stems from precisely this reason – we would be delighted if this study was repeated and expanded on in the future.  

 We calculated “citations per year” as the ratio between the total number of citations (before discontinuation plus after discontinuation) and the number of Scopus years. In the revised version of underlying data table 1 we have now added a box with a more detailed description to enable the readers to repeat our analysis. 

 However, we must point out that online data changes daily. Therefore, in order to reproduce the data to perfection, one would need to know for which one of the 317 journals that we studied - on which day through the duration of the study period we downloaded the data. The overall process took about a month as described in the paper. This issue may render the data not reproducible to the dot. However, at any time of examination, the overall trends should remain the same.  

**Comment 8.2:** *Some variable names and analysis are misleading, such as “Actual Pubmed”, described in methods section as “inclusion in PubMed” and in table 6 as “main database indexing”. It does not reflect whether the journal is currently indexed in PubMed, but may in some cases only indicate that a single article is present in PubMed or selected citations, due to their deposit in PMC (eg. “Advanced Materials Letters“).*

**Reply:** Thank you for pointing out this omission. We have now revised both the manuscript and underlying data table 1 to specify that we collected data on the inclusion of articles in PubMed. We also changed the title of Table 6 as follows: “Table 6. Discontinued journals’ current Open Access policy and the indexing of their articles in major databases”.

**Comment 8.3:** *Some data seem a bit bizarre… and information provided by the authors like “Citation before and after the date of discontinuation were manually counted based on either the Scopus journal overview or the downloadable tables made available by Scopus upon request (see source data)” (p.3) did not allow us to double check some numbers that were weirdly extreme, and potential typos. Some counts of the number of citations seem erroneous, leading to an aberrant number of citations per document for journals like "Mental Health in Family Medicine" (80 citations per document before discontinuation) or "Pharmacognosy Reviews" (170 after).*

*Other example of bizarre data: according to “underlying data table 1” the journal “Advanced material research” has been indexed for 10 years (from 2004 to 2014) and has received during this period only 3 citations. However, after having been delisted from the Scopus database, during a 6 year period (2014 to 2019), it has received 13875 citations. Any thoughts on how/why this could have happened?”*

**Reply: **Bizarre or not, the data is what it is. Here we describe how the data was collected in detail:

1. We searched the name of each journal using the Scopus database “Sources” page ( https://www.scopus.com/sources.uri?zone=TopNavBar&origin=searchbasic), then we opened the page of the journal and selected “view all documents”. 
2. At the following page, we checked the box “All” to select all the documents and then clicked “View citation overview”.
3. At this stage, two options may appear: a) a page with a line chart reporting the number of citations per year, or b) a flag inviting a request for a citation overview download due to the large size of the overview which does not allow on-site display as a line chart.
4. In the a) situation, we manually summed the citations displayed. In the b) situation, we inserted in the request form an email address and Scopus sent us back a .csv file for each request, containing the requested data.

As this detailed description add little to the main text of the paper we have not added it to the description of the methods. 

 We wish to reiterate the point made in our previous response – that data collection was not completed in a single day. The data for this study required accessing several sources for each paper, documentation of different variables from each source and performance of individual calculations. For this reason, we report the time frame during which the data was collected (24th January - end of February 2020).

 Citations are continuously updated as they grow and new journals are indexed in the database, so at any time other than the exact time we downloaded the data, the number of citations would be slightly different than ours.

 Specifically, with regards to the examples given for “bizarre” data, we have re-checked these items and our replies are as follows: 

1. Our table 1 did not and does not state “ *80 citations per document before discontinuation*” for Mental Health in Family Medicine. We report a total of 175 documents, with 213 citations before discontinuation and 14123 after discontinuation. Thus, 1.22 citations per document before discontinuation and 80 after discontinuation. 
2. Regarding Pharmacognosy Reviews - We are grateful to the reviewer for picking up on this typo - In the table, the number of citations before discontinuation was erroneously written as ‘43451’ rather than ‘4345’. This led to the number of 170 citations per year for the period before discontinuation. We have corrected the resultant calculations. We have also re-checked the database for additional typos (none were found). The main findings of the manuscript did not change after this correction. Nonetheless thank you for pointing out the mistake.
3. Regarding Advanced Material Research - we have re-checked the data and confirm that it is correct. We do not have an explanation for the huge difference between the period preceding and succeeding discontinuation.

 

 Minor comments:

**Comment 1:** *Abstract:*

*Background: “contains the largest number of abstract and articles…” -> "One of the largest" could be better, some databases are bigger than Scopus (Google Scholar and Dimensions, see https://doi.org/10.1016/j.joi.2018.03.0061 or https://arxiv.org/abs/2004.14329)*

**Reply: **Modified as suggested.

**Comment 2:** *Background: The term “publication concerns” may not be clear to everyone.*

**Reply:** Thank you for this comment. As this is a Scopus term, we cannot modify it. We have added quotation marks to make this clear to the readers.

**Comment 3:** *Background: “These journals remain indexed and can be cited.” This sentence is confusing. The articles published before the exclusion remains indexed and their citations continue to be taken into account but new issues of the journals are not indexed any more.*

**Reply: **Thank you for pointing out this misnomer. We have substituted ‘journals’ for ‘previously published articles’.

**Comment 4:** *Methods: The use of the term “discontinued” both for DOAJ (Results) and for journal publication (Background) is confusing. Should we say “excluded” or “delisted*”?

**Reply: **The term ‘discontinued’ is that used in the Scopus database. The label ‘coverage discontinued in Scopus’ is also displayed on the discontinued journals’ page. The downloadable list of journals whose coverage has been discontinued is also named by Scopus as ‘Discontinued sources from Scopus’. 

 As it is important that the labels used in the manuscript remain consistent with official labels and definitions, we felt we could not change the term ‘discontinued’.

**Comment 5:** *Results: “317 journals were evaluated” but next sentence states ninety-three percent of the journals (294/318)” -> typo for 318*?

**Reply:** Thank you. It was indeed a typo which has been corrected. Once again, thank you for the scrupulous review which has improved our paper. 

**Comment 6:** *Results: “the mean number of citations per year after discontinuation was significantly higher than before, and so was the number of citation per document”. Unclear whether the median difference of 64 is per journal, or cumulative across all “discontinued” journals? What are “documents”? Do you mean “articles”? or are there any other types of publication?*

**Reply: **We calculated the median number of cumulative citations across all discontinued journals per year of coverage and defined it as ‘Citations per year’. We calculated the median number of cumulative citations across all discontinued journals per document (‘Citations per document’). We did not give a subjective definition of ‘document’ but included all the indexed documents provided by the Scopus database. We have added this detailed description in the methods section of the paper, and we also specified the calculations performed in underlying table 1.

**Comment 7**: *Conclusions: it’s unclear how the conclusion regarding “predatory journals” is drawn. Also, we don’t think the career advancement are “artificial”, they are real! Although maybe “undue”?*

**Reply**: As a result of this comment we have modified the conclusions to state as follows: “Journals whose coverage in Scopus has been halted for publication concerns continue to be cited. This paradox may influence scholar metrics, potentially prompting career advancements and promotions. Further studies are needed, also investigating the journals discontinued from Scopus using the criteria “outlier performance – radar”, particularly effective in flagging potential predatory journals. Countermeasures should be taken to ensure the validity and reliability of Scopus metrics for both journals and authors due to their importance for scientific assessment of scholarly publishing. Creative thinking is required to resolve this issue without punishing authors who have inadvertently published good quality papers in a failing or predatory discontinued journal.”

**Comment 8**: *Introduction:*

*“publications from no longer indexed journals may not be removed retrospectively … hence articles … could remain part of the database7” p.3 -> this conditional statement seems to contradict abstract which categorically states “These journals remains indexed” as well as the author’s conclusion “we propose that CSAB could apply these measure case-by-case”. Reference 7, linked with statement, was not helpful to clarify.*

**Reply: **Thank you for pointing out that the language in this sentence requires improvement. 

 We have revised this to read more succinctly: 

 “The list of the discontinued sources is publicly available and is updated approximately every six months ^6^. However, articles published in journals that were discontinued and are no longer indexed, are probably not removed from the Scopus database. 

 It has been claimed…”     

**Comment 9**: *Methods:*

*“Independently collected by eight of the authors in pairs”: not very clear: two by two, or checked by two different people independently?*

**Reply**: Agree. We specified that four pairs of authors independently collected the data (i.e. two people independently collected the same quarter of the data. The entire database is the result of eight people collecting the data). 

**Comment 10:** *“the year of our data collection”: more precision maybe?*

**Reply: **Agree. We changed **“**the year of our data collection” with “2020”.

**Comment 11:** *Results:*

*Why were data from 31 journals not retrieved? What was the problem?*

**Reply: **The journals were not found on Scopus database using the search tool.  This is now also stated in the paper.

**Comment 12:** *Table 1: Interestingly, none of these journals (discontinued from Scopus for publication concerns) have been published by Elsevier. Could there possibly be a selection bias? An interesting option could be to check if Elsevier’s journals that are still in Scopus might have been discontinued from others sources (DOAJ, WOS), and on which grounds?*

**Reply: **We agree that this would be an interesting finding. In this study, we focused on journals discontinued by Scopus for ‘publication concerns’, and we afterwards checked whether they had also been delisted from DOAJ. Should the data mentioned by the reviewers regarding WOS be available online, it may worth further investigation. Thank you for this important insight.

**Comment 13**: *Table 1: Maybe the table could be enhanced to provide information about whether other journals by that publisher are still included in Scopus or not? Eg. 39 journals discontinued from Scopus were published by Academic journal Inc. Are there any other journals by this publisher still in Scopus?*

**Reply**: The relation between the publisher and de-indexing of articles in Scopus after discontinuation is an important question that should be addressed in further research. We aimed to provide a snapshot of the effect of ongoing article availability, rather than explore publisher and/or Scopus policies associated with journal discontinuation.

**Comment 14**: *Table 3: don’t need 2 decimal precision in %.*

**Reply**: This has been changed in accordance with the reviewer’s request. 

**Comment 15**: *Table 3: Subject area are repetitive in Scopus, and a journal can have more than one, while this table and underlying data mention only 1 per journal, presumably the first appearing in Scopus? If so, probably worth indicating and better to remove the percentages in table 3 falsely suggesting mutually exclusive categories?*

**Reply**: Indeed the reviewer is correct. The table caption now reads “First subject area as displayed in Scopus. Note: a journal may have more than one subject area”.  

**Comment 16**: *Table 5 and results (text): unclear where the median difference of 64 comes from? Or the 0.4?*

**Reply**: Thank you for asking. These are the results of the Wilcoxon matched-pairs signed rank test. We removed the reference to the table to avoid misunderstandings. The results differ slightly after corrections made following previous reviewers’ comments. The raw data are available in underlying data table 1 and the analyses can be easily recreated by the readers.

**Comment 17**: *Table 5: total number of citations does not match number of citation before and after discontinuation. Any thoughts on how/why this could have happened? A note of explanation about that would be useful.*

**Reply**: Again- we are grateful for the reviewers’ sharp eye. We rechecked the data and found a typo: The number of citations after discontinuation was reported as 607621 when it should have been 607261 (this can be seen in our underlying data table 1 and in the main text). We have corrected this in the new version of the manuscript.

**Comment 18**: *Citations by year in table 5: The number of years before and after the journal is removed from Scopus is very different, the average is more than 9 years before and 4 years after (median of 8 and 4 respectively) which makes the comparison in Table 5 not relevant. Indeed, the number of citations per year is higher during the 2 or 3 years following the publication of the article and decreases sharply with time (DOI:10.1371/journal.pone.01537302) so that the ratio of citations per year also decreases if a larger number of years is used.*

**Reply**: This is probably true. Although some papers may undergo resurgence this is probably not common. However, we found no better way of coping with the issue of the different number of Scopus coverage years across journals. And furthermore, this “decay” is likely to be fairly consistent across all journals both before and after discontinuation. As we also added to the study limitations the lack of a control group of non-discontinued journals, we have also inserted a word of caution regarding our results. 

**Comment 19**: *Distribution of articles: of the 317 journals analysed, 5 contain more than half of the articles concerned by this question. This very inhomogeneous distribution means that the statistical analyses and the percentages per journal do not take this kind of distribution into account.*

**Reply**: We used non-parametric tests to reduce the effect of non normal distribution of data on our findings precisely for this reason. We also present IQRs and ranges to be more informative.

**Comment 20**: *Page 5, first paragraph: table 2 should probably read table 5?*

**Reply**: We have removed this reference to table 2 in order to accommodate a previous reviewer’s comment (Comment 16).

**Comment 21**: *Page 5, 2nd paragraph: In 243 case (243/317)… is useless here. Maybe the authors meant 76.6% (243/217)*

**Reply**: This has been amended. Thank you. 

**Comment 22**: *Table 6: maybe a good idea to separate the “positive” facts (being indexed in Pubmed, WoS or Cabell’s whitelist) from the negative ones (Beall’s list, Cabell’s blacklist DOAJ discontinued.)*

**Reply**: This was a good idea. We have changed the order of the items in Table 6 accordingly.

**Comment 23:**

*Discussion:*

*p.7 Unclear why the term “ghost journal” suddenly appear and how it is defined.*

**Reply**: We modified the phrase; the text now reads “The fact that discontinued journals might help to move up in academia is a relevant issue, and has inspired the vignette depicted in Figure 2: “Discontinued journals may inflate authors’ metrics lifting them unnaturally and effortlessly.”

 We decide not to remove the term ‘inflate’ from Figure 2 caption as the figure is an allegorical and so intentionally exaggerated representation of the phenomenon.

**Comment 24**: *p. 7-8 “Of greatest concern is our finding that many of the discontinued journals display predatory behaviors in claiming to be open access” -> do you mean “displaying… are claiming”? to our understanding the article does not say that open access systematically means predatory. According to ref 9 et 22, the large majority of DOAJ indexed journals were not found in Beall’s list or Cabell’s Blacklist.*

**Reply**: We have changed the phrase “Of greatest concern is our finding that many of the discontinued journals display predatory behaviours in claiming to be open access” to “Of greatest concern is our finding that many of the discontinued journals display predatory behaviours in claiming to be open access without actually being indexed in DOAJ.”

**Comment 25**: *p. 8: “Such journals” unclear: predatory journals or OA journals?*

**Reply**: ‘Predatory’. This is now written. 

**Comment 26**: *The authors highlight that a limitation of their methodology is that they have included the year of discontinuation in the period “after discontinuation”, which could have led to overestimations. Then why not present the 2 analyses with the year of discontinuation included in the period BEFORE and in the period AFTER discontinuation, so that the reader can check for him-herself what bias this has induced?*

**Reply**: We mentioned the possibility of overestimation in order to be entirely honest. However, our impression, just from eyeballing the data during collection, was that this would not lead to much of a change. More importantly, in the early stage, we planned no such analysis and therefore did not collect the data that would be required to do this analysis. At this stage performing such an analysis practically requires that the data be recollected in near entirety again which is no simple task. 

**Comment 27**: *A mention of or comparison with other databases’ practices with regards to removing journals for indexing could be interesting. Do their approaches differ from Scopus’?*

**Reply:** This question again is one of policy and therefore not in the scope of our study. The reviewers’ comments indeed present much food for thought in terms of future research. 

**Comment 28**: *Conclusions:*

*Proposals are missing to solve the problem addressed and to avoid the stigmatization of the authors of the "suspect" articles. For example, a new open peer-review for articles published within X months before the journal's exclusion would be a possibility.*

*Maybe another idea would be to flag published articles that have been published in journals that are not indexed anymore “NB: this article was been published in YEAR, in a journal that has encountered publication concerns in YEAR”*

**Reply**: We appreciate the reviewers’ proposals and have now added them to the discussion. Solutions should also address metrics and citations deriving from these journals. We have therefore added that while this may be an immediately implementable temporising measure, additional thought should be dedicated to address of these aspects as well while maintaining fairness.

**Comment 29**: *References:*

*Reference 5 URL should be* *https://www.elsevier.com/__data/assets/pdf_file/0004/891058/ACAD_LIB_SC_ART_Importance-of-high-quality-content_WEB.pdf*

**Reply**: Corrected.

**Comment 30**: *Link in reference 12 does not work properly due to a superfluous space in the middle.*

**Reply**: Corrected.

**Comment 31**: *Reference style is not harmonized (cf. 1st § of methods section makes 4 references to the same underlying data table 1 (ref number 12 in the reference list, 2 of whom are not correct and should refer to source data, the rest having various citation style).*

**Reply**: Now harmonised.

**Comment 32**: There might be some mix up in references: ex. §4 on page 3 lists ref 6-8. Is it possible it should read 5, 7-8 instead?

**Reply**: We again salute the work done by the reviewer. An additional critical eye is always helpful. We have re-reviewed the references to ensure that no additional issues exist, but are of course willing to make any additional corrections if such are identified. 

**Comment 33**: The literature review would benefit from additional references to complete or contrast with the author’s findings: ex. doi:10.3390/publications80200173 that concludes that “articles published in predatory journals have little scientific impact.”

**Reply**: Thank you. We have now added this reference and discussed it. 

**Comment 34**: *Auto-citations: There are different ways to increase a researchers’ number of citations or H-index. Publishing in a “predatory journal” may be one of them, but auto-citation is also one. Of the 30 references cited at the end of this paper, 11 (37%) are auto-citations (citation of a reference including at least on author of the present paper), 7 (23%) are articles from others, and the remaining 12 were websites.*

**Reply**: Auto-citations are indeed an issue in the literature. However, in this case, we wish to highlight several points that make this comment moot: 

1. The reviewer included in their count three references: Underlying data Table 1: Standardized data extraction form with data collected [previously Ref. n° 12], Extended data Appendix 1 [previously Ref. n° 25] and Extended data Table 1. [previously Ref. n°26], that are study materials. F1000research policy explicitly requires adding supplementary study materials and all the underlying data (e.g. databases) in the reference list. Consequently, the real percentage of self quotations is actually lower.  
2. This manuscript has a long list of authors, many of which have published (1) together (2) on this topic. Hence the probability of citing previous research by these authors is high. Is the reviewer suggesting that researchers never base their newest work on their previous work?  Experts that consistently research any field of research are likely to work together and to cite their own work - each step supports the next. 

Despite the nature of this comment and our concern that it may detract from the quality of our paper, we have removed some of the references. The authors of this paper have no need of self citations – for promotion or any other purpose. 

**Comment 35**: *Typo/language*

*In Underlying data table 1: last column title - DOAJ instead of DOAH ?*

**Reply**: Corrected.

**Comment 36**: *p.3 §3 “journal are no longer be indexed” -> “journal are no longer indexed”*

**Reply**: Corrected.

**Comment 37**: *p.3 §5 “still they can get” -> “they can still get”*

**Reply**: Corrected.

# Peer review: Reviewer response for version 1

**DOI:** 10.5256/f1000research.26314.r63789

**Published:** 2020-06-16

**Authors:** Johann Mouton

**Affiliations:** 1 Centre for Research on Evaluation, Science and Technology (CREST), DST/NRF Centre of Excellence in Scientometrics and Science, Technology and Innovation Policy, Stellenbosch University, Stellenbosch, South Africa

This is a very relevant study in the growing scholarship around predatory publishing. It is one of the first studies that look at how predatory or at least questionable journals that have been delisted from citation database continue to have a presence in academia. More specifically, the paper asks the very important question why databases like Scopus (and others) continue to track the citations of journals that have been removed. This creates a distortion at many levels, including at the individual publication profile level.

 I am happy to recommend indexing of this paper as it is (some minor grammatical editing is required).

Is the work clearly and accurately presented and does it cite the current literature?

Yes

If applicable, is the statistical analysis and its interpretation appropriate?

Yes

Are all the source data underlying the results available to ensure full reproducibility?

Yes

Is the study design appropriate and is the work technically sound?

Yes

Are the conclusions drawn adequately supported by the results?

Yes

Are sufficient details of methods and analysis provided to allow replication by others?

Yes

Reviewer Expertise:

Bibliometrics; scholarly publishing; science policy; sociology of science

I confirm that I have read this submission and believe that I have an appropriate level of expertise to confirm that it is of an acceptable scientific standard.

# Response

**Published:** 2020-08-13

**Authors:** Andrea Cortegiani

**Affiliations:** University of Palermo, Italy

Dear reviewer,

 We are glad to submit a revised version of our manuscript, previously entitled ‘ **Inflated citations and metrics of journals discontinued from Scopus for publication concerns: the GhoS(t)copus Project** **’**. The comment you provided was helpful in revising and improving the manuscript.

 We hereby provide our reply.

 Best regards,

 Andrea Cortegiani, MD

 On behalf of co-authors

 _______________________________________________________________________

**Response to** **Reviewer 1** (Johann Mouton, Centre for Research on Evaluation, Science and Technology (CREST), DST/NRF Centre of Excellence in Scientometrics and Science, Technology and Innovation Policy, Stellenbosch University, Stellenbosch, South Africa)

**Comment**: *This is a very relevant study in the growing scholarship around predatory publishing. It is one of the first studies that look at how predatory or at least questionable journals that have been delisted from citation database continue to have a presence in academia. More specifically, the paper asks the very important question why databases like Scopus (and others) continue to track the citations of journals that have been removed. This creates a distortion at many levels, including at the individual publication profile level.*

*I am happy to recommend indexing of this paper as it is (some minor grammatical editing is required).*

**Reply**: We thank the reviewer for the comment. We have now edited the manuscript and corrected errors and typos.
